# Spirituality alleviates the burden on family members caring for patients receiving palliative care exclusively

**DOI:** 10.1186/s12904-020-00585-2

**Published:** 2020-06-03

**Authors:** Paula Menis Vigna, Isac de Castro, Renata Rego Lins Fumis

**Affiliations:** 1 Department of Psychology, Hospital da Luz, São Paulo, Brazil; 2 Division of Nephrology and Molecular Medicine, Department of Medicine, University of São Paulo School of Medicine, São Paulo, Brazil; 3 Intensive Care Unit, Hospital Sírio-Libanês - São Paulo, Rua Dona Adma Jafet, 115 - Bela Vista, São Paulo, SP 01308-050 Brazil

**Keywords:** Spirituality, Family caregivers, Emotional burden, Palliative care

## Abstract

**Background:**

Spirituality can give meaning to life, providing support and guidance in complex situations. Despite its importance in palliative care, the role of spirituality for family caregivers of patients under exclusive palliative care has not received enough attention in the literature. We aimed to address the correlation between spirituality and the emotional burden of family members of patients under exclusive palliative care.

**Methods:**

This transversal study was conducted in a tertiary private teaching hospital, in São Paulo, Brazil. The study comprised family members of patients receiving palliative care exclusively. Only one caregiver who cared for the patient for at least 2 months was invited to participate. Family members answered the following questionnaires: WHOQOL spirituality, religiousness and personal beliefs (SRPB), Zarit Burden Interview (ZBI) and Self-Reporting Questionnaire (SRQ-20). They were excluded if patients were residing in a Long Stay Institution. Continuous variables were expressed by median and quartiles and analyzed with the Kruskal-Wallis test with Muller-Dunn post-test adjusted by Bonferroni or with the Mann-Whitney test for two groups. We used multivariable linear regression to identify independent predictors of caregiver burden.

**Results:**

A total of 178 family members were interviewed in a median of 8 [4–13.25] days after patient admission. Almost 40% of families presented high score of burden. Faith and Meaning in Life were the facets that scored the highest, with a median of 4.50 [4.00–5.00] for both facets. There was an inverse correlation between Zarit score and all of the WHOQOL-SRPB facets, indicating that the lower the spirituality, the greater the emotional burden. Inner peace was the strongest protective factor associated with burden.

**Conclusions:**

Psycho-socio-spiritual interaction can improve the coping ability of family caregivers of patients under exclusive palliative care, addressing a critical gap in the provision of holistic palliative care services.

## Key statements

- Family members with higher spirituality felt less burdened by the care of their loved ones.
- The health care team should systematically address spirituality in family members of patients under palliative care.

## Background

Spirituality may give meaning to life and enhance faith. Providing support and guidance in complex situations, spirituality and spiritual care are gaining increasing attention by many authors in the context of palliative care [1, 2]. Second only to treatment, religiousness and spirituality play a major role in both survivors and caregivers [3].

An interdisciplinary approach to palliative care in cancer patients is important for improving the social well-being of families and for alleviating their burden and psychological distress [4]. In the Intensive care unit (ICU) setting, a previous study suggests that conversations with families about the patients´ spiritual preferences, with specific reference to his or her wishes for end-of-life care, may be particularly useful. Spiritual support was associated with increased family satisfaction in the ICU, providing comfort, especially in the face of hardship [5].

Likewise, when at home, the patient-caregiver dyad also benefits from emotional and spiritual support [6], emphasizing the need to accurately measure spiritual outcomes in diverse palliative care populations [7]. Caregivers with less spirituality are at higher risk of burden, anxiety, and stress [8]. Caregivers of end-stage cancer patients with higher levels of intrinsic spirituality spend more time with the patients and are better protected against emotional distress [9]. Religiousness and faith help some caregivers cope with the suffering associated with the care of their relatives with advanced cancer [10].

Spirituality was defined as’ a dynamic and intrinsic aspect of humanity, through which people seek ultimate meaning, purpose and transcendence and experience relationships with themselves, with family, with other people, with the community, with society, nature and with the meaningful or “sacred” [11]. Spiritual care, that is, the support of health professionals who aim to improve spiritual suffering is still largely neglected in clinical practice, and few patients receive this spiritual assistance [12]. Family caregivers report high levels of spiritual suffering and unmet needs, particularly in the context of a life-limiting illness [13].

Surprisingly, spirituality has been scarcely studied at the end-of-life of medical care when one would expect it would matter the most. We hypothesized that, in patients under exclusive palliative care, there would be an inverse relationship between the spiritual well-being and the burden of caregivers. To this end, we assessed the correlation between spirituality and the emotional burden of family members of patients hospitalized under exclusive palliative care.

## Methods

### Setting

This transversal study was conducted in a tertiary private teaching hospital, Hospital da Luz, in São Paulo, Brazil. It is a 226-bed hospital, where the Palliative Care Team treats an average of 38 patients per month. The palliative care team consisted of physicians, nurses, psychologists, and respiratory therapists. Although the hospital had no Chaplaincy service, patients and their families received spiritual support on demand, and the family member could bring spiritual care specialist such as chaplains and pastoral care providers, if they wanted.

The institutional review board (IRB) “Comitê de Ética em Pesquisa da Sociedade Beneficiente de Senhoras do Hospital Sírio-Libanês” reviewed and approved the study (HSL 2016–03, protocol number 1.392.591 and IRB of Hospital da Luz, protocol number 1.439.581). The written informed consent was obtained from all participants.

The study comprised family members of patients under exclusive palliative care. Only one caregiver who cared for the patient for at least 2 months, usually a family member defined by the patient, was invited to participate. Patients were eligible for the Exclusive Palliative Care Program if they had a chronic, progressive, life-limiting disease / condition in which treatment would not modify or stabilize the disease; when there was no possibility of new treatments, and when the chronic disease or condition was associated with a functional profile of 40% or less on the Karnofsky Performance Status Scale (KPS).

Family members under 18 years of age, those without cognitive conditions to understand the questionnaire, and family members of patients residing in a Long Stay Institution were excluded.

A single researcher (P.M.V.), an experienced psychologist, interviewed family members in a quiet place for approximately 30 min. Interviewees answered the Self-Reporting Questionnaire (SRQ-20), the Zarit Burden Interview (ZBI), and the WHOQOL spirituality, religiousness and personal beliefs (SRPB).

The SRQ-20 is a screening tool developed by the World Health Organization specifically for the low and middle income countries, primary healthcare setting, widely used in several countries including Brazil. We used it to identify minor psychiatric morbidity. Each of its 20 items is rated as absent or present by the respondent (scores 0 and 1, respectively). The score’s range can vary from 0 to 20. Higher scores represent higher levels of mental distress. We used the cutoff score of 7/8, based on a Brazilian study to differentiate possible cases of mental distress [14].

The reduced version of ZBI scale was comprised of seven items, enabling to assess the burden of caregiver in the context of palliative care, as well as issues related to self-care and loss of social and / or family role. The score can range from 7 to 35 points. Emotional burden is identified if the score is equal to or higher 17 points [15].

The WHOQOL-SRPB scale has 32 questions covering quality of life aspects related to spirituality, religiousness, and personal beliefs. The 32 questions are divided into 8 facets, each comprising 4 items, namely “Connectedness to a Spiritual Being or Force”; “Meaning of Life”, “Awe”, “Wholeness and Integration”, “Spiritual Strength”, “Inner Peace”, “Hope”, and “Optimism and Faith”. Responses to each item are rated on a 5-point Likert scale, ranging from 0 [‘not at all’] to 5 [‘an extreme amount’]. Each facet score is derived by averaging the score obtained from the responses to the 4 questions comprising that particular facet [16]. The WHOQOL-SRPB was developed to assess spirituality and have been used in clinical research that measure spirituality [17, 18]. All Palliative Care interview scales were previously validated in Brazil [14–16].

### Data collection

For each patient, the following information was collected: age, gender, religion, marital status, schooling, income, main diagnosis, reason and time of inclusion in the Exclusive Palliative Care Program, and KPS. The following variables of the family caregiver were collected: age, gender, marital status, Charlson index, relationship with the patient, years of schooling, income, time taking care for the patient, extra-caregiver activities, religion, spirituality, emotional burden.

### Statistical analysis

Categorical data were expressed by absolute (n) and relative frequency (%) and analyzed with the Pearson χ2. Continuous variables were expressed by median and quartiles and analyzed with the Kruskal-Wallis test with Muller-Dunn post-test adjusted by Bonferroni or with the Mann-Whitney test for two groups. We used multivariable linear regression to identify independent predictors of caregiver burden. Results are expressed by beta values and standard errors. Variables with a significant association with burden in the Spearman Correlation (*p* < 0.1) were included in the multivariable model. We used a forward stepwise selection procedure to define the final model. A *p* value < 0.05 was considered statistically significant.

## Results

From March 2016 to February 2017, 261 patients were admitted to the Exclusive Palliative Care Program and were eligible for this research. Of these, eight refused to participate, 38 met exclusion criteria, and 37 patients had left the hospital (hospital discharge or death) before being approached by the researcher. A total of 178 family members were interviewed. Families were interviewed a median of 8 [4–13] days after admission.

Most family members were women (79.21%), married (65.7%), and offspring of the patients (52.2%). The majority (79.78%) of family members reported that they take care of the patient with a frequency of 3 to 5 times a week, and only 10.1% cared for the patient single-handedly. Regarding family health, a great part of family members (73.6%) presented at least one comorbidity according to Charlson index. Almost 40% of families presented high score of burden.

Table 1 shows the distribution of Family member’s characteristics according to Zarit score measure of burden. We found a significant association between level of burden and female gender, age of family caregivers, higher schooling, and more time spent per day caring for the patient. Patients’ marital status and their religion were associated with family caregiver burden (Table 2).

**Table 1:** Family member’s characteristics according to Zarit score

Family members variables (***n*** = 178)	n (%)	Zarit score Median [CI]	***p*** value
Gender			0.030
Female	141 (79.2)	15 [10–20]	
Male	37 (20.8)	13 [9–15]	
Age range (years)			0.057
20–29	11 (6.2)	20 [14–24]	
30–39	15 (8.4)	16 [9–20]	
40–49	25 (14)	16 [13–21]	
50–59	59 (33.1)	15 [10–20]	
60–69	39 (21.9)	12 [9–18]	
≥ 70	29 (16.3)	13 [9–16]	
Age (categorized)			0.002
< 65 years	134 (75.3)	15 (11–21)	
≥ 65 years	44 (24.7)	12 (9–16)	
Marital status			0.108
Single	44 (24.7)	16 (12–23)	
Married	117 (65.7)	14 (10–19)	
Widower	6 (3.4)	12 (9–18)	
Divorced	11 (6.2)	13 (9–16)	
Schooling (categorized)			0.016
< 8 years	46 (25.8)	13 (9–16)	
≥ 8 years	132 (74.2)	15 (11–21)	
Religion			0.365
Catholic	109 (61.2)	14 (11–20)	
Evangelical	25 (14)	14 (9–20)	
Spiritualist	27 (15.2)	15 (10–19)	
None	13 (7.3)	16 (12–18)	
Others	4 (2.2)	8 (7–14)	
Personal income (dollars/month)			0.838
Até $300,00	60 (33.7)		
$300,00–$800,00	56 (31.5)		
$800- $1300,00	30 (16.9)		
> $1300,00	32 (18)		
Frequency of taking care of the patient, days per week			0.041
1–2 per week (a)	11 (6.2)	12 [8–15]	(*p* < 0.05 a versus c)
3–5 per week (b)	25 (14)	13 [9–18]	
6–7 per week (c)	142 (79.8)	15 [11–21]	

Kruskal-Wallis test with Muller- Dunn post-test or Mann –Whitney

**Table 2:** Patients characteristics according to family members’ Zarit score

Patients variables (***n*** = 178)	n (%)	Zarit score Median [CI]	***p*** value
Gender			0.969
Female	113 (63.5)	14 [10–20]	
Male	65 (36.5)	14 [10–19]	
Age range (years)			0.819
Until 39	5 (2.8)	14 [13–14]	
40–49	13 (7.3)	15 [11–22]	
50–59	17 (9.6)	15 [12–21]	
60–69	21 (11.8)	15 [11–18]	
70–79	38 (21.3)	14 [9–18]	
80–89	55 (30.9)	15 [9–22]	
90–99	29 (16.3)	13 [10–16]	
Marital status			0.036
Single (a)	19 (10.7)	12 (8–18)	(*p* < 0.05 d versus a)
Married (b)	80 (44.9)	14 (9–20)	
Widower (c)	70 (39.3)	15 (11–20)	
Divorced (d)	9 (5.1)	18 (14–20)	
Religion			0.049
Catholic (a)	122 (68.5)	14 (10–20)	(*p* < 0.05 e versus b,c)
Evangelical (b)	30 (16.9)	15 (12–21)	
Spiritualist (c)	14 (7.9)	15 (12–18)	
None (d)	6 (3.4)	13 (8–18)	
Others (e)	5 (2.8)	7 (7–9)	
Personal income (dollars/month)			0.782
Until $300,00	72 (40.4)	14 [10–9]	
$300,00–$800,00	75 (42.1)	14 [9–20]	
$800- $1300,00	22 (12.4)	15 [12–22]	
> $1300,00	9 (5.1)	14 [11–19]	
Discharge			0.788
Death	85 (47.8)	14 [9–20]	
Home	70 (39.3)	14 [11–20]	
Hospice	23 (12.9)	15 [9–19]	
Diagnosis			0.690
Cancer	90 (50.6)	15 [11–20]	
Neurologic disease	58 (32.6)	14 [10–18]	
Lung diseases	11 (6.2)	13 [7–23]	
Heart diseases	9 (5.1)	21 [9–25]	
Others	10 (5.6)	18 [10–20]	

Kruskal-Wallis test with Muller- Dunn post-test or Mann –Whitney

Table 3 shows Spearman correlations between WHOQOL-SRPB facets and the Zarit score. Note that that there is an inverse correlation between Zarit score and all of the WHOQOL-SRPB facets, indicating that the lower the spirituality, the greater the emotional burden.

**Table 3:** WHOQOL-SRPB facets according to Zarit score

WHOQOL-SRPB Facets	Zarit Total score Correlation coefficient	*P* value Two sided significance
Spiritual Connection	−0.18	0.018
Faith	−0.25	0.001
Spiritual Strength	−0.29	< 0.001
Meaning in Life	−0.36	< 0.001
Wholeness & Integration	−0.41	< 0.001
Admiration	−0.42	< 0.001
Hope & Optimism	−0.43	< 0.001
Inner Peace	−0.46	< 0.001

Family caregivers scored high on all facets of the WHOQOL-SRPB questionnaire. The facets with highest score were Faith and Meaning in Life, and Inner Peace was the one with the lowest score (Table 4).

**Table 4:** Measures of WHOQOL-SRPB facets

WHOQOL-SRPB facets	Median [IQ]	Min- Max	Median [IQ] Score 100 ^**a**^
Spiritual Connection	4.25 [3.75–4.56]	1.0–5.0	85 [75–91.25]
Meaning in Life	4.50 [4.00–5.00]	1.25–5.0	90 [80–100]
Admiration	4.25 [3.75–4.75]	1.5–5.0	85 [75–95]
Wholeness & Integration	4.00 [3.50–4.50]	2.0–5.0	80 [70–90]
Spiritual Strength	4.37 [4.00–5.00]	2.0–5.0	87.5 [80–100]
Inner Peace	3.75 [3.25–4.25]	1.5–5.0	75 [65–85]
Hope & Optimism	4.00 [3.50–4.50]	1.0–5.0	80 [70–90]
Faith	4.50 [4.00–5.00]	1.0–5.0	90 [80–100]
SRPB Total	16.62 [15.0–18.0]	8.8–20.0	83 [75–90]

^a^Scored with the original WHOQOL-100 spirituality facet. (Cronbach Alpha = 0.957)

Table 5 presents the independent predictive factors for emotional burden identified in the multivariable analysis. Inner peace was the most important protective element of burden. Note also that the family caregiver schooling was an important risk factor.

**Table 5:** Predictors factors for Burden

Category	Non-standardized coefficients	Standard Error	Standardized coefficients	***p*** value
Spiritual connection	**1.49**	**0.55**	**0.37**	**0.007**
Inner peace	**−2.35**	**0.58**	**−0.53**	**< 0.001**
Time per day caring to the patients	**0.25**	**0.06**	**0.25**	**< 0.001**
Family schooling	**0.68**	**0.10**	**0.49**	**< 0.001**
Age of family caregiver	**0.07**	**0.03**	**0.24**	**0.031**
KPS at baseline	**0.09**	**0.04**	**0.16**	**0.020**

We found a positive correlation between the Zarit score and the SQR-20 score, showing that the increase in burden was associated with higher scores of psychiatric disturbance (*r* = 0.57, *p* < 0.001). Higher spirituality as measured by the SRPB score was associated with lower Zarit scores (*r* = − 0.45, *p* = 0.001) and also with lower SQR-20 score (*r* = − 0.37, *p* < 0.001), that is, spirituality has an inverse relationship with burden and psychiatric disturbances.

Regarding SQR-20, 30.3% of family members scored above seven points, a finding indicative of some psychiatric disturbance (mean score 5.21 ± 3.87 points, ranging from 0 to 20 points). Women had higher SQR-20 scores compared to men (5.61 ± 3.94 versus 3.70 ± 3.21, *p* = 0.007), and younger family caregivers (≤ 60 years) scored higher than older ones (6.13 ± 4.20 versus 3.62 ± 2.56, *p* < 0.001).

We found a high Cronbach Alpha = 0.957 in WHOQOL-SRPB questionnaire; Zarit scale Cronbach Alpha = 0.848 and Cronbach Alpha of SQR-20 = 0.83.

## Discussion

According to the World Health Organization – “Palliative Care improves the quality of life of patients and their families facing the problem associated with life-threatening illness, through prevention and relief of suffering by means of early identification and impeccable assessment and treatment of pain and other problems, physical, psychosocial and spiritual”. Despite empirical evidence of spiritual support as part of palliative care, spirituality remains insufficiently addressed by the medical system [19].

Family caregivers of palliative cancer patients are usually heavily burdened and suffer with their changes of life. They often need to reduce their social life and bear a strong impact on emotional distress. Spirituality has been depicted in some studies as a protective factor of emotional burden mainly in the case of family members of critically ill patients and those with chronic diseases [20–23].

The most important finding in our study was that family members with higher spirituality felt less burdened to take care of their loved ones under exclusive palliative care. The other significant finding was that all WHOQOL-SRPB facets were important in reducing the burden. Interestingly, family members attributed to the facets Faith and Meaning in Life the highest scores compared to the other facets of spirituality and Inner Peace was the strongest predictor of protection against burden.

Several studies have found lower levels of anxiety and depression in advanced cancer patients with higher levels of spiritual wellbeing [24–26]. The multidimensionality of spiritual well-being, which includes constructs such as meaning and faith, have a potential impact on QOL for lung cancer patients, notwithstanding their religious affiliations [27]. More directed to cancer patients in general, the focus of these studies was not on exclusive palliative care [24, 26–28]. Our study extends these findings to family members of patients under exclusive palliative care and suggests that the role of inner peace in illness are important to the spiritual experience of those who are taking care of seriously ill patients. These findings support the recommendations of The National Consensus Project for Quality Palliative Care (NCP) for attention to the religious/spiritual aspects of palliative care, such as performing a spiritual history and attending to spiritual concerns and needs by making referrals to chaplaincy and other spiritual supporters [29].

A complicated and multidimensional phenomenon, spirituality is an essential element connected to seeking meaning, purpose, and transcendence in life [30]. However, day-to-day spiritual practice was found to be potentially protective against burnout in patients with mental illness [31]. According to the recommendations of Puchalski et al., spirituality should be considered a ‘vital sign’ in patients. Just as pain is routinely screened for, so should spiritual issues be a part of routine care [32].

The question remains as how these needs should be assessed. Clinicians can provide spiritual care by asking open-ended questions as part of a social history. These questions can enhance spiritual and social understanding of the patient that is essential to the biopsychosocial-spiritual experience of illness [33]. Several questionnaires are available to assess spiritual needs [34, 35]. The assessment should be conducted with a great deal of care and consideration. Tools such as the FICA spiritual history tool (F - Faith and Belief; I – Importance; C – Community; A - Address in Care) help clinicians invite patients and families to share their spiritual or existential concerns as well as sources of hope and meaning which can help them cope better with their illness [36]. For example, The Spiritual Interests Related to Illness Tool (SpIRIT) was created by Taylor in 2006 in the USA to investigate the spiritual needs prevalent in patients with cancer and their caregivers [37]. The stem of all the questions in this self-report tool started with “How important is it now to … ”, and at the end of each group of questions, an item asked: “How important it is to have my (or my loved one) nurse help me satisfy these spiritual interests?” [34, 37]. The Daily Spiritual Experience Scale (DSES), developed by Dr. Lynn Underwood captures a set of experiences that can play an important role in people’s lives. The DSES evaluates how often people experience, in their routine daily lives, feelings such as the God’s presence, strength and comfort in the religion or the spirituality, connection to all of life, love for others, admiration for nature, inner peace, gratitude for blessings and desire to be close to God [38].

Family members caring for loved ones with chronic disease such as cancer are at high risk for psychological distress, which may impair their quality of life due to elevated burden. Previous studies point out that caregivers who experience low spiritual well-being have a poorer Quality of Life and more problematic intrapsychic aspects of personality, such as low acceptance of their own emotions and failure to be in contact with their own feelings [39, 40]. It is important to identify the problems and address the spiritual needs of caregivers in order to provide support for their spiritual well-being and reduce their suffering.

In this study, having spent intense and uninterrupted hours of care was among the predictors of burden. This finding suggests the need for supporting family members who spend many hours per day taking care of their loved ones. Attention should also be directed to the financial situation of caregivers. We found that 73.6% of caregivers had to give up their jobs at least partially, which has been associated with major personal and social problems [41].

Our study has several limitations. It was carried out in a single center and had a small sample size. Additionally, our population consisted of in-patients only. Previous studies reveal a jeopardized quality of life and mental health burden in family members of outpatients with advanced cancer [42]. This population refers to patients receiving exclusive palliative care. Our results should not be generalized to a broader population of family members of patients under palliative care. This study used the reduced version of ZARIT and it is not possible to find out if the reduction of items did not interfere with the original use of the questionnaire. Finally, although spirituality and religiousness are distinct constructs, our sample consisted mainly of Catholics. Spirituality has different meaning and roles in different cultures, countries and different religious beliefs. Future cross-cultural and cross-religious investigations could clarify the relationship between spirituality and caregiver burden.

## Conclusions

Our study showed that the psycho-socio-spiritual interaction can improve the coping ability of family caregivers, addressing a critical gap in the provision of holistic palliative care services. Spirituality in the context of patients under exclusive palliative care can help them cope and alleviate their burden.

## Data Availability

The data can be obtained under reasonable request from the corresponding author with permission from Renata Rego Lins Fumis, e-mail regolins@uol.com.br.

## Abbreviations

ICU: Intensive Care Unit
IRB: Institutional Review Board
HSL: Hospital Sírio-Libanês
KPS: Karnofsky Performance Status Scale
SRQ-20: Self- Reporting Questionnaire
ZBI: Zarit Burden Interview
WHOQOL: SRPB World Health Organization – Spirituality, Religiousness and Personal beliefs
QOL: Quality of Life
NCP: National Consensus Project
FICA: Faith and Belief; Importance; Community; Address in care
SpIRIT: Spiritual Interests Related to Illness tool
DSES: Daily Spiritual Experience Scale
